# Heat‐induced compounds development in processed tomato and their influence on corrosion initiation in metal food cans

**DOI:** 10.1002/fsn3.2376

**Published:** 2021-06-27

**Authors:** Elliot Dhuey, Hardy Z. Castada, Sheryl Barringer, Jojo Joseph, Christopher M. Hadad, Ken Ruffley, Melvin A. Pascall

**Affiliations:** ^1^ Department of Food Science and Technology The Ohio State University Columbus OH USA; ^2^ Department of Chemistry and Biochemistry The Ohio State University Columbus OH USA; ^3^ Kenneth J Ruffley LLC Cincinnati OH USA

**Keywords:** canned food, corrosion, dimethyl sulfide, nitrate(s), tomato(es), volatiles

## Abstract

Selected ion flow tube mass spectrometry (SIFT‐MS) and ion chromatography (IC) were used to investigate the presence of volatile and nonvolatile compounds in canned tomatoes and in the polymeric lining before and after retorting the cans. This allowed us to observe if these compounds contributed to corrosion and the migration of iron and tin compounds from the cans to the tomatoes. Diced Roma tomatoes and other simulant treatment groups were sealed in two‐piece tinplated cans (controls in glass jars), retorted at 121℃ for 30 min, then stored at 49℃ for 50 days. Results showed that thermal degradation of amino acids in the tomatoes gave rise to volatile methyl sulfides and nonvolatile nitrogenous compounds which were subsequently sorbed by the can lining. SIFT‐MS showed a 20‐fold increase in dimethyl sulfide concentration. Inductively coupled plasma (ICP‐MS) results showed fourfold and 16‐fold increases in iron and tin compounds, respectively, that migrated from the metal to the tomatoes as a result of acid and electrolyte interactions.

## Graphical abstract

The process of washing, peeling dicing, canning and retorting of the tomatoes.

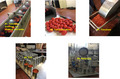

## 1 INTRODUCTION

Tomato (*Solanum lycopersicum)* is a warm‐season crop that is sensitive to frost. When harvested, most of the crop is processed into canned whole tomatoes, juices, paste, ketchup, chili, and barbecue sauces, as examples, in order to provide year‐round availability of the commodity (AgMRC, 2018; Razdan & Mattoo, 2006). However, processed tomato is considered one of the most aggressive products with potential to initiate corrosion when packaged in metal containers (Razdan & Mattoo, 2006). Metal cans have emerged as an excellent tool to extend the shelf life of food because these containers have high barrier to gases, vapors, light, filth, and microorganisms. At the same time, the complexity of canned food products greatly varies from one another and many natural and added ingredients are known to contribute to the initiation of corrosion in metal packaging. Corrosion is defined as a chemical reaction between a metal and its environment to form derivative compounds of the metal. This involves the transfer of an electrical charge across the boundary between the metal surface and the environment. Components in food that are known to accelerate this corrosion include oxygen, pigments, nitrates, sulfur compounds, sodium chloride, and trimethylamines (Robertson, 2012).

The tomato fruit has nearly 400 identified volatile compounds derived from various enzymatic or kinetic reactions of larger molecules (Petro‐Turza, 1986). Dimethyl sulfide is one of the most prevalent volatile compounds found in heat‐processed tomato‐based products (Maarse, 2017). Dimethyl sulfide is largely formed from the breakdown of methyl methionine which is formed via biosynthetic reactions during the development of the tomato fruit (Mudd & Datko, 1990). Tomatoes also contain many nonvolatile compounds including nitrates. A portion of the nitrates found in tomatoes come from the fertilizers used in the growing soil (Gould, 1992; Rao & Puttanna, 2000). Nitrogenous fertilizers in the soil are readily absorbed by the tomatoes in the form of ammonia, nitrate, and nitrite compounds during harvesting if the fruit is contaminated with soil prior to washing. The natural low acidic character of tomatoes gives it the capability of oxidizing these nitrates and nitrites into compounds with corrosion potential. Nitrates are also known as depolarizers which can contribute to the detinning of the metal in food cans (Albu‐Yaron & Feigin, 1992; Palmieri et al. 2004).

Because sulfur and nitrogen compounds found in canned foods are known to accelerate corrosion in the metal container, methionine, and nitrate were targeted as possible contributors to the corrosion occurring in this study (Ninčević Grassino et al., 2009; Razdan & Mattoo, 2006). Furthermore, compounds that are known to interact with polymers, like sulfur compounds, may have the potential to complex with and alter the polymer that comprises the corrosion‐resistant lining of the cans. When a molecule is sorbed by a polymeric matrix, it can plasticize the chains of the polymer and this can facilitate an exponential uptake of additional molecules by the polymer (Comyn, 2012).

In order to retard the progress of corrosion in metal containers used to package tomatoes, it is necessary to understand the types of compounds that are associated with these foods when they are heated under pressure within sealed metal cans. This must be followed by locating the parts of the cans where the corrosion is occurring. This information is essential because corrosion occurring in the headspace of a can is an indication of the action of volatiles, while those occurring in the lower body of the can indicate the action of higher molecular weight compounds. When corrosion occurs in a can filled with food, chemical reaction between the metal surface and the electrolytes gives rise to a metal complex that has potential to dissolve in an aqueous medium. When this occurs in canned tomato products, it could increase the tin and iron content of the product and if it is excessive, an unsafe situate could develop.

The objectives of this study were as follows: (1) to identify and quantify the volatile and nonvolatile compounds associated with processed tomatoes under retort conditions; (2) to understand which of these compounds were responsible for breaching the protective lining and caused corrosion in retorted cans; and (3) to quantify metallic compounds from the corroded areas of the cans that migrated to the packaged tomatoes.

## 2 MATERIALS AND METHODS

### 2.1 Materials and ingredients

Two types of packaging materials were tested in this study: BPA‐free epoxy‐lined metal cans and 237 ml Ball Regular Mouth Mason glass jars. The metal cans were 211 × 400 drawn and wall‐ironed (D&I) in size and supplied by PPG Industries Inc. (Milford, OH). The glass jars were purchased from a local grocery store in the Columbus, Ohio area. Roma tomatoes (*Solanum lycopersicum*) and salt (sodium chloride) were purchased from a local food supermarket in the Columbus, Ohio area. Red mature tomatoes of uniform size were chosen and stored at room temperature for 3 days after being purchased. Citric acid, calcium chloride, sodium nitrate, methionine, and sulfonium methyl methionine used in this study were purchased from Sigma‐Aldrich, Co.

### 2.2 Experimental design

**FIGURE 1:**
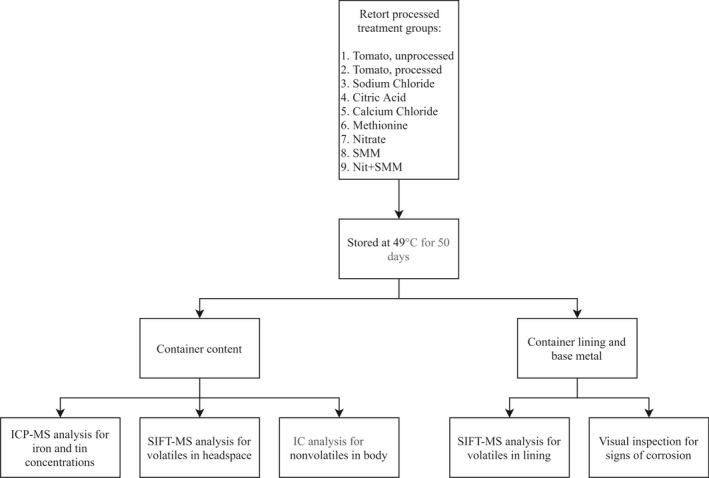
Experimental design illustrated

The experimental design is illustrated in Figure 1. Canned diced tomatoes were stored for a 50‐day period, and then, the contents were tested for volatile and nonvolatile compounds at select time points of the storage period. The volatile contents were analyzed to determine the effects of thermal processing on the development of compounds in the headspace of the metal cans. The nonvolatile contents were analyzed to determine the effects of thermal processing on the development of compounds in the body of the metal cans. These compounds were monitored throughout the 50‐day storage period. Additionally, volatile compounds that sorbed to the internal polymeric lining of the can during the storage period were monitored. To accomplish this, the lining in each can was removed then analyzed for the presence and concentrations of the organic volatiles. Because of the potential loss of corrosion protection provided by the lining, the cans were visually inspected throughout the 50‐day storage period for signs of corrosion. The visible signs of corrosion were then associated with the concentrations of iron and tin that migrated from the metal surface into the contents of the container.

### 2.3 Sample preparation, processing, and storage

The tomatoes were peeled, diced, canned, sealed, and retorted using the facilities in the Food Processing Pilot Plant in the Department of Food Science and Technology at The Ohio State University, Columbus, Ohio. The whole tomatoes were washed by submersion in a tank of potable water that was agitated by a circulation pump. The washed tomatoes were sorted to remove damaged and oddly shaped fruits. They were then soaked in 18% sodium hydroxide and 0.1% Faspeel (which is a proprietary mixture of fatty acids used as a peeling aid) (Collinsville, IL) solutions for 30 s at 88℃ in order to soften the outer skin. They were then placed on a conveyor belt fitted with rotary rubber disks that provided a scrubbing action to the tomatoes as they passed under a low‐pressure water spray. This resulted in the complete removal of the tomato skins. The peeled tomatoes were then diced into 1.27‐cm^3^ cubes. One‐third of the entire batch of diced tomatoes was then reduced in size to a pumpable slurry using a W.J. Fitzpatrick Model D Comminuting Machine (Chicago, IL) equipped with a 1.9 cm screen. The slurry was then pumped to a tubular heat exchanger. This was heated using steam to reach a hot break temperature of 88℃. The juice was extracted using a Chisholm‐Ryder Model CLE‐360‐D28 screw type extractor (Kalamazoo, MI) with a 5 mm mesh screen.

A 278‐gram aliquot of the diced tomatoes was transferred to a 211 × 400 two‐piece can. To this was added 36 grams of tomato juice, leaving a headspace of 7.14 mm in the can. A total of 32 cans were similarly prepared. The cans were then closed using a Model#6 American Can Seamer (Greenwich, CT). After similarly filling the 237 ml glass jars with the diced tomatoes and juice, they were tightened by hand to a torque level that prevented leaking (9 Nm). A total of 4 of these glass jars were prepared and used as controls. All samples were adjusted to a pH of 3.55 using citric acid prior to filling and sealing the cans and glass jars. The hermetically sealed cans were sterilized in a Dixie Canner Still Retort (Athens, GA) at a hold temperature of 121℃ for 30 min. The glass jars were retorted separately from the metal cans because of the difference in heat penetration properties of glass when compared with that of tin‐plated steel. For the glass jars, they were heated at 121℃ for 40 min to achieve commercial sterility. The heating times and temperatures were selected based on recommendations from the USDA, to ensure that the cold spot of the containers filled with the diced and juiced tomatoes reached a minimum temperature of 121℃ (USDA, 2015).

The diced tomato formulation comprised diced tomato (87.94% w/w), tomato juice (11.40% w/w), sodium chloride (0.42% w/w), citric acid (0.19% w/w), and calcium chloride (0.05% w/w). The recipe of the canned diced tomatoes was modified to selectively eliminate each ingredient, thereby creating various treatments of the canned product (shown in Table S1). Either the tomato (diced or juiced), citric acid, calcium chloride, or sodium chloride was selectively left out from each treatment group.

Additional treatment groups were added with simulants in distilled water adjusted to pH 3.55 with citric acid. These treatment groups include s‐methyl methionine (SMM) or SMM combined with sodium nitrate (Nit + SMM). The methionine group was formulated with citric acid and sodium chloride in a solution of distilled water. The nitrate group was formulated using the same chemicals as the methionine group. To prepare the methionine (15 ppm) samples, 0.90 grams of L‐methionine was added to 6 L of a buffer solution that consisted of 11.4 grams citric acid, 25.2 grams sodium chloride, and 3 grams calcium chloride in 6 L of distilled water. To prepare the nitrate (25 ppm) samples, 0.15 gram of sodium nitrate was added to 6 L of another batch of the buffer solution.

A total of four controls were used in this experiment. The control groups contained all the ingredients in the diced tomato formulation (diced tomato, juice, sodium chloride, citric acid, and calcium chloride). In order to compare volatile, nonvolatile, and metal concentrations, the first control group was retort processed in glass jars, the second control group was retorted processed in metal cans, and the third control group was not heat treated but filled in glass jars to be tested at day 0. The fourth control did not contain any ingredients and was unprocessed can linings that were used to compare with processed can linings which contained the ingredients from each treatment group as shown in Table S1.

To obtain a hermetically sealed can, the double seam was required to have a body hook between 1.90 and 2.16 mm, a cover hook between 1.78 and 2.29 mm and an overlap >1.14 mm. The average seam width measurement of the tested cans used in this study was 2.87 mm. The average body hook was 1.93 mm, cover hook was 1.91 mm, and overlap was 1.23 mm. After sealing, all samples were shaken to ensure proper mixing of the ingredients. The retorted canned diced tomato samples and other treatment groups were then stored at 49℃ prior to testing.

### 2.4 Quantification methodology

During the storage period, volatile and nonvolatile compounds in the canned contents and the can linings were analyzed using a SYFT Voice200 selected ion flow tube‐mass spectrometry (SIFT‐MS) instrument (Syft Ltd.) and a Thermo Scientific Dionex ICS‐1500 Ion Chromatography system, respectively. Iron and tin metal complexes that migrated from the metal cans to the internal contents were analyzed using Thermo Fisher Element 2 Inductively Coupled Plasma Mass Spectrometry (ICP‐MS) (Thermo Fisher).

### 2.5 Analysis of volatile concentrations of treatment group contents and linings

The SIFT‐MS instrument was used for the headspace analysis of the volatile organic compounds at the parts‐per‐billion level by volume (ppb_v_) in the test containers. After retorting but prior to the SIFT‐MS analysis, all samples were stored for 50 days at 49℃. Each treatment group was opened and tested on days 0, 3, 6, 10, 20, 30, 40, and 50.

For quantification of the volatile compounds in the canned diced tomato treatment groups, the mixtures were homogeneously blended using a Waring, Dynamic Corp. commercial blender (Torrington, CT, USA) for 30 s at the highest speed setting. From each sample, a 5‐gram aliquot was transferred to a 500 ml Pyrex media storage bottle. Each bottle was capped with an open top screw cap fitted with an airtight silicone septum and incubated at 70℃ in a water bath for 5 min to allow for headspace equilibration. For quantification of the volatile compounds in the can lining, the lining was removed from the headspace region of each test can after the can was cut into four quadrants. From each can, a 0.006‐gram mass of the lining was transferred to a 500 ml Pyrex media storage bottle. Each bottle was sealed and incubated at 90℃ in a water bath for 5 min. Before the analyses, all media glass storage bottles and septa were incubated at 100℃ for 12 hr to remove all traces of residual volatile compounds.

The headspace of each test bottle was analyzed using the selected ion monitoring (SIM) mode of the SIFT‐MS instrument. The headspace sampling was performed using a 14‐gauge 3.8 cm long passivated sampling needle connected to the inlet port of the SIFT‐MS instrument. It was inserted into each test sample bottle through the septum of the bottle cap. Prior to introducing the samples into the equipment, a 5 ml aliquot of a 50℃ HPLC grade water was injected into the system in order to clean the high‐performance inlet tube of the instrument. A blank analysis was done before and after each test and used to zero the equipment and minimize carry‐over effects. The room air was also scanned between each sample test in order to also zero the instrument. The scan duration was 1 min.

The analyses targeted sulfur and acid compounds as well as other chemicals of interest in tomatoes. Table S2 summarizes the information used to identify the 22 volatile organic compounds of interest in the headspace of the bottles. The data collected were expressed as concentrations in ppb_v_ of the compounds. Each treatment group showed the average of 6 data points (2 batches and 3 observations per batch).

Additionally, to determine if sulfonium methyl methionine (SMM) thermally degraded to dimethyl sulfide, increasing levels of SMM were added to 2 ml distilled water in a 50 ml Pyrex jar fitted with a septum in its cap. The samples (in replicates of 4) were heated at the same retort processing conditions as the tomatoes (121℃ for 30 min) in the closed system and then analyzed for dimethyl sulfide by the SIFT‐MS.

### 2.6 Analysis of nonvolatile concentrations of treatment group contents

An ion chromatography (IC) instrument was used to determine the nitrate concentrations in the tomato samples. Prior to the analysis, the samples were stored at 49℃. The canned tomatoes were tested on days 0, 3, 6, 10, 30, and 50 of storage, while those in the glass jar were tested on days 0, 20, and 50. The unprocessed tomato samples were stored at −80℃ in glass jars prior to analysis. The samples were tested from 2 batches with 3 replicates per batch. All samples were homogenized using the commercial blender for 30 s at the highest speed setting. The blended samples were centrifuged at 5,000 r/min for 30 min using a Sorvall Legend X1 Centrifuge manufactured by Thermo Scientific (Waltham, MA). The aqueous supernatant was collected and recentrifuged a second time at the same conditions as previously reported. The supernatant was removed and then diluted by a factor of 8 with distilled water.

The ion chromatography instrument was fitted with a Dionex IonPac AS22 anion exchange column (250 mm × 4 mm) and an AG22 Guard column. The mobile phase was 1.4 mM sodium bicarbonate ⁄ 4.5 mM sodium carbonate in water, and it was filtered with a 0.45 µm nylon membrane at a flow rate of 1 ml⁄ min. The total analysis duration time was 20 min, and the injected volume was a 5 ml aliquot.

### 2.7 Analysis of iron and tin concentrations of treatment group contents

To measure the migration of the tin and iron compounds from the metal can to the packaged tomato, a Thermo Fisher Element 2 ICP‐MS (Bremen, Germany) instrument was used. All chemicals used in the analysis were of analytical‐reagent grade and were not purified further. Deionized water processed by an 18 MΩcm^‐1^, Millipore Milli‐Q‐Plus water purifier (Bedford, MA) was used in all testing on the ICP‐MS. The standard solutions for the iron and tin analyses were obtained from dilutions of stock solutions which included the single element iron (CPI International) and the single element tin (CPI Internal). From these, standard curves were plotted for iron and tin in concentrations of 10, 25, 50, 100, 150, and 200 ppb. Colbalt‐59 (CPI International) was used as an internal standard in all sample solutions at 10 ppb. The stock solutions were diluted with a solution containing 2% HNO_3_ and 0.05% HF. Iron‐56 and Tin‐120 were selected for monitoring the concentrations of iron and tin migration from the packages to the tomato contents. The concentrations of iron and tin were determined from the standard curves by plotting the intensities versus the concentrations of iron and tin from the external standards and interpolating them with the intensities for the test samples.

The tomato products from the test cans and the glass jars (controls) were tested on days 0, 10, 30, and 40 and days 0, 20, and 40, respectively, after being held at a storage temperature of 49℃. The treatment groups containing SMM, and sodium nitrate with SMM, were both tested on days 0, 20, and 40 of the storage period. The samples were tested from 2 batches with 3 replicates per batch. The samples were homogenized using the commercial blender for 30 s at the highest speed setting. An aliquot of 2 grams from each sample was weighed using either plastic spoons or micro pipetted into 50 ml polypropylene plastic tubes. Aliquots of 5.0 ml of a 69% HNO_3_ and 2.5 ml a 30% H_2_O_2_ were added to each tube including the blank tube. The tubes were loosely capped for 10 min to allow for partial digestion and then tighten before transferring to a digestor. The tubes were then incubated at 105℃ for 2.5 hr in a DigiPREP MS digestion system manufactured by SCP Science, Inc. After digestion, the tubes were cooled to room temperature (23℃ ± 2). The samples were then diluted with a solution of 0.05% HF and 10 ppb of cobalt (as an internal standard) in 50 ml of deionized water.

The samples were analyzed by ICP‐MS where the intensities of ^56^Fe and ^120^Sn were used to determine the concentrations of iron and tin in the tested samples. The samples were introduced into the instrument at 100 microliters/min (μL/min) through a concentric PFA micronebulizer into a PFA double pass spray chamber. The ICP RF power was 1,250 watts. A rinse of 2% HNO_3_ was scanned before each analysis and between the samples to clean the loading tube. A standard check and calibration blank were scanned after every 10th test sample reading. The standard solutions were scanned from low to high concentration to create a calibration curve. The results were collected using the Thermo ELEMENT software. Each sample was automatically measured five times and the average concentration calculated. The concentration of each test sample time point was averaged between 2 batches and 3 replicates per batch.

### 2.8 Statistical analysis and data interpretation

The statistical analyses of the data were carried out using an IBM® SPSS® Statistics Program Version 24 Software. Linear regression was used to compare the significant effects of each treatment group on the rates of the tin and iron migration during the storage period. An analysis of variance (univariate and multivariate) was used with post hoc tests of Fisher's Least Significant Difference (LSD) at an alpha level of 0.05 to investigate the following: (1) the significant effect of heat processing on the concentrations of the volatile and nonvolatile compounds before and after processing the tomatoes and during the storage period; (2) the volatile compounds that had significant interaction with the epoxy coating during the storage period; and (3) the significant effect that each treatment group had on the migration of tin and iron compounds from the walls of the cans to the tomato product during the storage period.

## 3 RESULTS AND DISCUSSION

### 3.1 Effect of tomato retort process on volatile compounds concentration

The concentrations of selected volatile compounds in the processed tomatoes and in the protective lining of the cans were identified and quantified using a SIFT‐MS method. Table 1 shows the mean concentrations of the selected compounds in the tomatoes before and after the retorting process in glass packaging. After retorting, there was a significant increase (*p*‐value <.05) in sulfur compounds such as dimethyl sulfide (DMS), dimethyl disulfide, and dimethyl trisulfide in the tomatoes when compared with the unprocessed samples. Table 1 shows that the concentration of DMS increased approximately 20‐fold after the retorting process. Additionally, methanol, ethanol, acetaldehyde, and acetone also increased in concentration in the headspace of the packages after retorting. Among the selected acid compounds, acetic acid increased significantly (*p*‐value<.05) after retorting.

**TABLE 1:** Concentrations (ppb_v_) of selected volatile compounds in the tomatoes before and after retort processing in glass containers

Volatile compounds	Concentration of analytes in 3 grams of sample (ppb_v_)		
	Unprocessed	Processed	*p*‐value
Sulfurs			
Dimethyl disulfide	17	28	.004
Dimethyl sulphide	259	5,208	<.001
Dimethyl trisulfide	30	62	<.001
Methyl mercaptan	80	70	.272
1‐propanethiol	19	21	.309
2‐isobutylthiazole	18	20	.439
Acids			
Hexanoic acid	5	2	.016
Hexyl acetate	2	1	.541
Butanoic acid	94	83	.252
Acetic acid	65	118	<.001
Others			
Methanol	176,373	184,909	.590
Ethanol	17,996	17,042	.308
Furaneol	9	11	.286
Furfural	7	140	<.001
Hexanal	273	83	<.001
Phenylacetaldehyde	4	79	<.001
(E)−2‐hexenal	29	58	<.001
(E)−2‐octenal	40	0	<.001
(E)−2‐pentenal	135	87	<.001
Acetaldehyde	2,332	4,156	<.001
Acetone	630	2,145	<.001
Ammonia	173	194	.500

Note

Values in Table 1 are expressed as the mean of 2 batches by 3 replicates per batch.

To determine which compounds were sorbed from the tomato by the lining of the cans after retorting, changes in the concentrations of the selected volatile compounds in the lining were examined before and after the retorting process. The results in Table 2 show that the DMS significantly (*p*‐value <.05) increased in the lining after the retort processing. All other compounds found at high concentrations in the tomato content were not found to be significant (*p*‐value >.05) in the lining (Table 2).

**TABLE 2:** Concentrations (ppb_v_) of selected volatile compounds in the can lining before and after retort process

Volatile compounds	Concentration of analytes in 6 mg of can lining (ppb_v_)		
	Unprocessed	Processed in tomato	*p*‐value
Sulfurs			
Dimethyl disulfide	0	0	.168
Dimethyl sulfide	0	62	<.001
Dimethyl trisulfide	0	3	.002
Methyl mercaptan	0	0	.017
1‐propanethiol	0	1	.540
2‐isobutylthiazole	0	0	<.001
Acids			
Hexanoic acid	−1	0	.143
Hexyl acetate	−2	0	.002
Butanoic acid	−1	−1	.954
Acetic acid	−3	−3	.932
Others			
Methanol	8	44	.081
Ethanol	29	78	.206
Furaneol	0	0	.092
Furfural	0	1	.002
Hexanal	1	2	.005
Phenylacetaldehyde	1	1	.689
(E)−2‐hexenal	0	1	<.001
(E)−2‐octenal	0	0	.345
(E)−2‐pentenal	0	1	.024
Acetaldehyde	12	11	.804
Acetone	1	6	.251
Ammonia	−4	16	.125

Note

Values in Table 2 are expressed as the mean of 2 batches by 2 replicates per batch.

Comyn (2012) reported that DMS has the potential to complex with and alter polymers such as those used in the lining of cans. When this occurs, DMS plasticizes the polymer's chains resulting in the uptake of corrosive compounds through the polymeric matrix (Comyn, 2012). A study by Kontominas et al. showed that sulfur and other compounds in canned fish products were sorbed by the polymeric lining, causing pores and cracks which resulted in corrosion and the migration of tin and iron into the package contents (Kontominas et al., 2006).

### 3.2 Effect of storage period on concentrations of volatile compounds in various treatment groups

Table 3 shows changes in the concentrations of the selected volatile compounds in the tomato treatment groups during the storage period at 49℃. The concentrations of the sulfur‐containing compounds in the tomatoes showed a continuous decrease during the 50‐day storage period. Additionally, the concentrations of the aldehyde compounds and butanoic acid also decreased during the storage time. Visible signs of corrosion occurred during the storage period and associated with it was the formation of ferrous sulfide and stannous sulfide. Nagu et al. (2018) and Singer (2017) reported that when these complex interactions occur, they decrease the volatility of the sulfur compounds. To confirm that these compounds were originally from the tomato fruit and not from the added ingredients, their concentrations in each of the ingredients were measured. This included the citric acid, calcium chloride, and sodium chloride treatment groups as shown in (Table S3). The results show that none of the identified compounds were also detected at the same levels as found in the tomato treatment group.

**TABLE 3:** Concentrations (ppb_v_) of selected volatiles in the tomato treatment group during storage at 49^O^C

Volatile compounds	Concentration of analytes in 3 grams of sample (ppb_v_)							
	Day 0	Day 3	Day 6	Day 10	Day 20	Day 30	Day 40	Day 50
Sulfurs								
Dimethyl disulfide	19	13	13	9	11	12	13	12
Dimethyl sulfide	3,836	3,258	3,403	2,508	3,158	3,159	3,546	3,076
Dimethyl trisulfide	46	27	25	19	24	24	29	20
Methyl mercaptan	49	31	28	23	26	25	29	24
1‐propanethiol	27	20	20	17	22	21	25	21
2‐isobutylthiazole	15	9	8	6	8	7	9	6
Acids								
Hexanoic acid	0	2	2	1	2	2	3	2
Hexyl acetate	−1	0	1	0	1	0	0	1
Butanoic acid	68	58	53	45	43	37	42	32
Acetic acid	76	61	56	52	69	74	97	147
Others								
Methanol	136,369	96,608	97,194	79,675	90,670	92,142	104,443	91,277
Ethanol	14,563	12,948	12,505	11,070	11,841	11,077	13,051	11,232
Furaneol	6	3	3	3	4	4	6	5
Furfural	57	47	60	66	106	129	183	223
Hexanal	76	61	57	45	52	50	54	43
Phenylacetaldehyde	32	30	31	24	22	20	22	17
(E)−2‐hexenal	32	20	19	15	19	20	28	28
(E)−2‐octenal	−3	2	2	1	2	2	2	2
(E)−2‐pentenal	64	43	42	32	37	36	42	34
Acetaldehyde	2,640	1,853	1,707	1,240	1,228	1,070	1,120	908
Acetone	1,143	1,072	1,502	1,086	1,218	1,424	1,321	1,294
Ammonia	115	65	60	42	48	43	55	39

Note

Values in Table 2 are expressed as the mean of 2 batches by 3 replicates per batch.

Table S3 shows the results of the methionine treatment group which was tested to determine whether it contributed to the formation of the DMS. The results show that there was no detection of the DMS at day 0; however, a noticeable increase in the dimethyl disulfide and methyl mercaptan concentrations was detected during the storage time of this methionine treatment group. These results seem to indicate that the methionine reacted to give rise to the dimethyl disulfide during the storage of the retorted product. Table S4 shows similar results for the nitrate treatment group, in which the same level of methionine was added to the nitrate solution.

Farrow et al. (1970) reported that nitrates will react with tin (II) to oxidize it to the more stable tin (IV), where 1 mole of nitrate will be reduced to ammonia for every 4 moles of tin that will be oxidized. This will happen because ammonia was the principal nitrate reduction product at pH levels below 5.0. Additionally, the literature reported that nitrates will be reduced to nitrous oxide (N_2_O), nitric oxide (NO), hydroxylamine (H_3_NO), and ammonia/ammonium ions when exposed to low pH conditions (Mannheim & Passy, 1982). It seems reasonable to conclude that the nitrate treatment group would show increased levels of ammonia during the storage time in the presence of corrosion as described by (Farrow et al. 1970). However, the concentration of ammonia was not high enough in our study, as can be seen in the preceding tables. The low detection of ammonia might be due to its loss when the cans were opened to remove the contents after retorting. This concentration would also be expected to be low in cans that did not show or lacked substantial corrosion.

### 3.3 Comparison of DMS concentrations in SMM and tomato treatment groups content and lining during storage period

Williams and Nelson (1974) reported that the formation of DMS in tomatoes occurred due to the thermal degradation of methyl methionine. The following equation illustrates the mechanism proposed by Scherb et al. (2009) for the formation of DMS (1) caused by the thermal degradation of SMM (2).

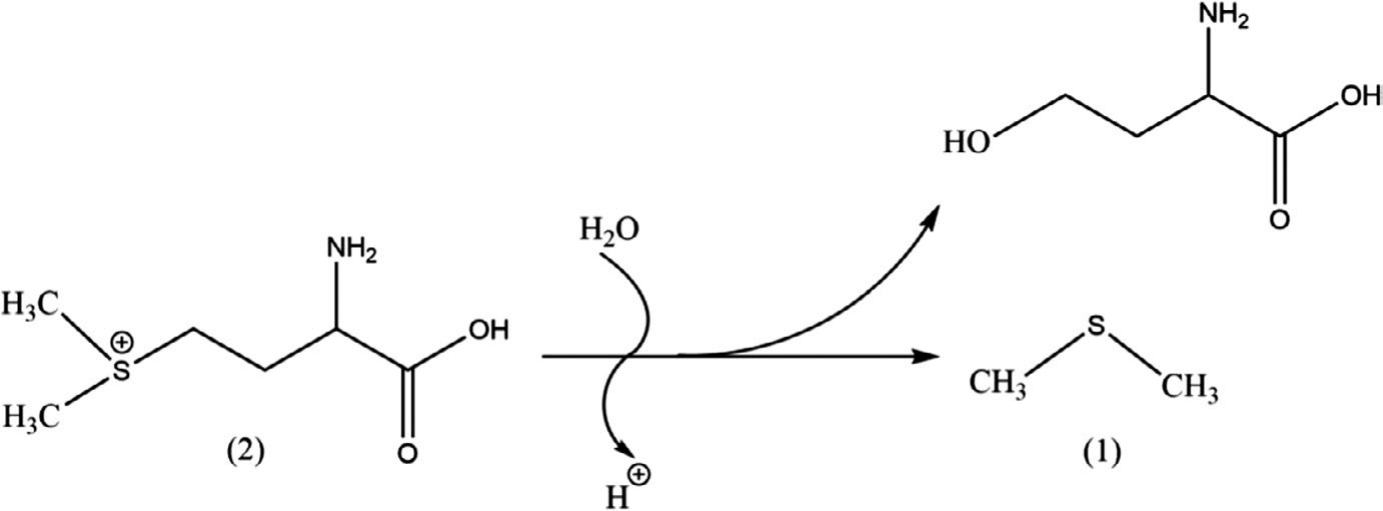

The equation shows that DMS concentration was directly proportional to increasing levels of the SMM after being heated for 30 min at 121℃ in an oven. Table S5 shows that the concentrations of the DMS gradually decreased in our study during the storage of the SMM treatment group. The initial high concentration of DMS seems to indicate that the degradation of SMM was responsible for its formation. Tables S6 and S7 show that the DMS was also detected in the linings of both tomato and SMM treated cans, respectively. The concentration of DMS in the SMM lining gradually decreased during the storage time, whereas the DMS showed no change in the tomato treated lining.

To gain a better understanding of the data generated, a post hoc LSD test was performed on the processed and unprocessed tomato and the SMM groups on day 0 and day 50 of the accelerated shelf life study. At day 0, the concentrations of the DMS in the tomato and the SMM treatment groups were not significantly different (*p*‐value >.05). However, both concentrations were significantly (*p*‐value <.05) higher than that of the unprocessed tomato group at the same time period. After 50 days of storage at 49℃, the DMS concentration in the SMM treatment group significantly declined from a mean of 3,390 to 19 ppb_v_, while the level in the tomato treatment group declined slightly (3,836 to 3,076 ppb_v_). These results suggest that the heat treatment had an influence on the DMS concentration.

A post hoc LSD test that was performed on the can lining of the processed and unprocessed tomato and SMM groups on day 0, 10, and 50 of the accelerated shelf life study in order to also better understand the trends in the data. At day 0, the concentration of the DMS in the can lining was significantly (*p*‐value <.05) different among the three groups. The lining of the unprocessed cans had no detectable levels of DMS, while 62 and 156 ppb_v_ of DMS were detected in the tomato treatment group and the SMM treatment group, respectively. Additionally, DMS concentrations in the linings of the tomato and the SMM treatment groups were significantly (*p*‐value <.05) different during the storage periods. At day 10, the concentration of the DMS in the lining increased to 166 ppb_v_ for the tomato treatment group, while it decreased to 89 ppb_v_ for the SMM treatment group. At day 50, the DMS concentration in the lining decreased for both the tomato treatment group (103 ppb_v_) and the SMM treatment group (0.60 ppb_v_). These results suggested that the rate at which the DMS interacted with the lining was slower for the tomato treatment group when compared with the SMM treatment group.

In summary, retort processing of tomatoes produces many volatile compounds with potential to interact with the polymeric lining of metal cans. Among the targeted volatile compounds in this study, the DMS was shown to have the highest concentration for significant interaction with the can lining. The formation of the DMS was due to the thermal degradation of methyl methionine which is naturally found in the fresh tomato plant [6]. The retort process provided sufficient heat to drive the reaction that converted methyl methionine into the DMS and the subsequent interaction of DMS with the polymeric matrix of the can lining. This interaction has the potential to form breaches in the lining which can allow sulfur and other acidic and potential corrosive compounds (penetrants) to diffuse through the polymer to the base metal. It is known that certain penetrants have the ability to plasticize the polymer, increase its segmental mobility, and increase the rate of diffusion of the substance through the polymer. Comyn (2012) confirmed this theory when he reported that increase sorption of a penetrant will decrease the segmental mobility of the polymer but increase the polymeric interactions. This interaction was observed by Kontominas et al. (2006) where canned fish resulted in sulfur induced black spots on the enamel surface of the package. These black spots were generated from ferrous sulfide while brown spots were formed by stannous sulfide. Other studies have found that sulfur compounds in fruits and vegetables can also contribute to metal corrosion, where the sulfur compounds are involved in the initial stages of corrosion (Charbonneau, 1997; Helwig & Biber, 1990). The SMM treatment group in this present study showed an initial increase in the DMS concentration (in both the tomato content and the lining of metal can), then the concentration decreased over the 50‐day storage period. This result might be closely related to previous observations on corrosions. These observations showed that as the can corroded, the sulfur compounds interacted with the tin and iron and caused a decrease in the observed volatility due to interaction with the metal surface microstructure. Nagu et al. (2018) reported that sulfur compounds at high temperatures in a wet environment will oxidize metal such as iron and the sulfur will be reduced to form ferrous sulfide in the following reaction scheme:

$${\text{Fe}}^{2+}+S^{2‐}\to \text{FeS}$$

### 3.4 Effect of storage period of tomato treatment group on nitrate concentration

**FIGURE 2:**
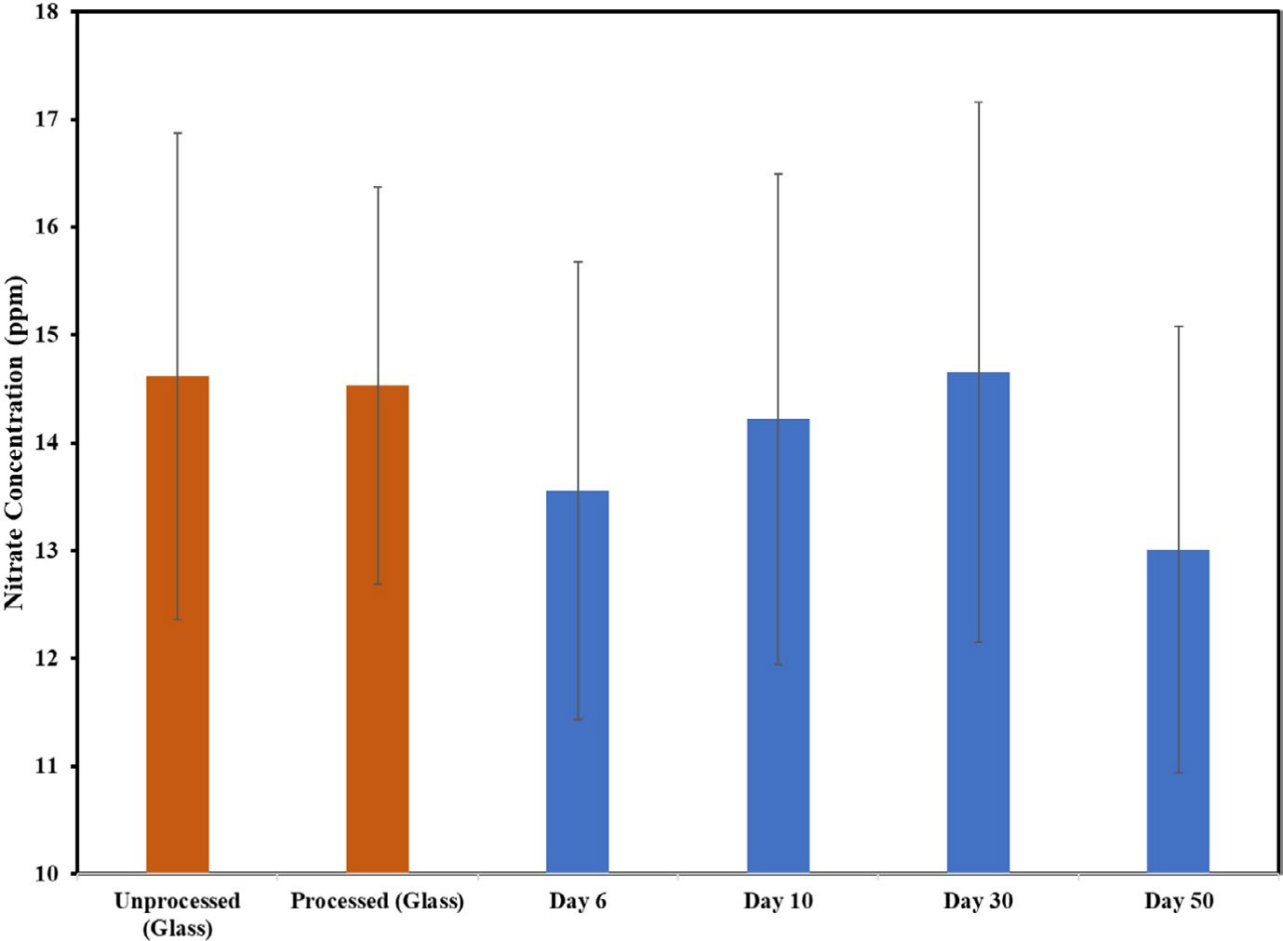
Nitrate concentrations of tomato processed in the glass jar and metal can during storage at 49 degrees C.

Figure 2 shows the concentration of nitrate in tomatoes processed in metal cans and glass jars (control). These were analyzed using an IC method during the 50‐day storage period. The statistical analysis showed that there were no significant differences between any of the treatment groups or time points (*p*‐value>.05). Palmieri et al., (2004) reported that nitrates can cause corrosion and become reduced to nitrite and ammonia when heated in canned tomato products. This has also been supported in studies done by Albu‐Yaron and Feigin, (1992), hence, why it was tested in our study. The concentrations in the samples in our study were relatively low, perhaps due to cultivation practices with the Roma tomatoes that were purchased for this research. Additionally, the storage period may not have been long enough to cause a greater decrease in the nitrate concentration. This can be seen as Figure 2 shows a decreasing ppm trend of nitrate concentrations as the storage period increases.

### 3.5 Effect of storage period of various treatment groups on iron and tin concentrations

Table 4 shows the concentrations of iron and tin in the tomato, SMM, and SMM+Nitrate treatment groups that were packaged in glass and metal containers. The iron and tin concentrations remained constant during the storage time in the control tomato glass jar group. These results show the natural levels of iron and tin found in the tomato itself because there was no expectation of migration of iron and tin from the glass packaging. Compared to the control group, the iron and tin concentrations in the packaged tomatoes in the metal containers were higher. The iron and tin concentrations both increased over time from day 0 to day 40 of the shelf life study, indicating that these elements originated from the metal packaging material.

**TABLE 4:** Concentrations of iron and tin of the various treatment groups during storage period

Sample	Average metal concentration (ppb)	
	[^56^Fe] ±*SD*	[^120^Sn] ±*SD*
Control (Glass Jar), Day 0	74.89 ± 8.88	10.05 ± 1.61
Control (Glass Jar), Day 20	72.58 ± 4.59	9.70 ± 1.69
Control (Glass Jar), Day 40	74.55 ± 5.54	11.58 ± 1.95
Tomato, Day 0	141.88 ± 18.81	22.19 ± 1.34
Tomato, Day 10	170.41 ± 24.43	63.03 ± 10.77
Tomato, Day 30	225.23 ± 14.38	93.10 ± 14.77
Tomato, Day 40	318.88 ± 60.69	163.98 ± 27.02
Tomato, Day 50	279.80 ± 91.37	135.69 ± 48.87
Distilled Water, Day 0	1.37 ± 0.25	1.19 ± 0.02
SMM, Day 0	2.31 ± 1.14	1.50 ± 0.15
SMM, Day 20	204.14 ± 46.43	1.63 ± 0.41
SMM, Day 40	215.38 ± 23.52	1.35 ± 0.02
SMM +Nitrate, Day 0	2.06 ± 0.69	1.48 ± 0.03
SMM +Nitrate, Day 20	201.86 ± 27.27	1.39 ± 0.02
SMM +Nitrate, Day 40	451.58 ± 97.91	1.40 ± 0.03

To understand the corrosion process that took place in this study, an observation of Table 4 shows the concentrations of iron and tin in the SMM treatment group compared with the SMM+Nitrate treatment group. The comparison of the two treatment groups with each other and against the control, contributed to an understanding of the impact of the SMM, in the absence of nitrate, on the can lining followed by the addition of the nitrate. Table 4 shows that the iron content increased from day 0 to day 20 in the SMM group. After day 20, the iron content showed a slight decrease, which indicated that the rate of iron migration may have slowed. When the nitrate was added to the SMM mixture, the iron concentration increased after day 20. These results seem to indicate that the nitrate increased the rate of corrosion and this in turn increased the rate of iron and tin migration from the cans to the packaged tomato product. This is illustrated in the following equations:

$$4\text{Sn}\to 4{\text{Sn}}^{++}+8e^{‐}$$

$$\text{Slow}:{\text{NO}}_{3}^{‐}+2e^{‐}+2H^{+}\to {\text{NO}}_{2}^{‐}+H_{2}O$$

$${\text{Fast: NO}}_{2}^{‐}+6e^{‐}+8H^{+}\to {\text{NH}}_{4}^{+}+2H_{2}O$$

No concentration change in the tin was seen during the storage period in either the SMM or the SMM+Nitrate treatment groups. These results seem to indicate that the SMM and nitrate did not affect the tin‐plate layer of the metal can but instead affected the steel layer. However, the tomato treatment group showed an increase in the concentration of tin during the storage period. Because the tomato group showed tin migration and the SMM and SMM+Nitrate groups did not, there might be another contributing factor that led to the tin corrosion in the canned tomatoes.

Multiple comparisons were conducted between tin concentrations of the treatment groups on day 0 and day 40. The tin concentrations on day 0 and day 40 during the storage of the cans were significantly different (*p*‐value <.05) between the tomato treatment group and both the SMM and SMM+Nitrate groups. Whereas, no differences in tin concentrations were found between the SMM and SMM+Nitrate treatment groups on day 0 and day 40 (*p*‐value >.05). This phenomenon can be best explained by the mechanisms of pitting corrosion which occurs in areas of imperfection in tin‐plated steel. This was seen using a scanning electron microscope where the results are shown in a subsequent paper. This could also happen if a breach in the polymeric lining occurs and the exposed tin‐plated layer experiences localized corrosion that exposed the steel layer. During pitting corrosion, the iron is more anodic than tin. Therefore, the tin does not corrode first, and the steel will have pitting corrosion in exposed areas that are tin‐free. This phenomenon often occurs with sulfur compounds which will result in a loss of cathodic protection from tin due to tin sulfide interactions. This reduces the rate of tin dissolution and provides no electrochemical protection to the steel from the tin‐plate coating (Robertson, 2012). Pits were observed in the cans of this present study and have been reported in other studies with canned tomato products (Albu‐Yaron & Feigin, 1992; Perring & Basic‐Dvorzak, 2002).

To determine the effect of each treatment on the corrosion in the metal packaging, the rate of iron migration for each treatment group was compared during the 40‐day period using a linear regression model. The linear fit was significant (*p*‐value <.05) for each treatment group, indicating a linear upward trend in the iron migration over the 50‐day storage period. When compared to the SMM+Nitrate treatment group (11.238), the tomato and the SMM treatment groups (4.495 and 5.327, respectively) had a lower rate of iron migration. Because the same concentrations of SMM and nitrate were also found in the tomato itself, one could expect that the rate of iron migration would have been the same in the SMM and SMM+Nitrate treatment groups. However, the data show that the concentrations of iron at day 40 in the SMM+Nitrate treatment group (451.58 ppb) were higher than that of the tomato treatment group (318.88 ppb). Therefore, the availability of the nitrates and the SMM to interact with the surface of the metal, which lead to an increase in the rate of corrosion and chemical migration, was greater than that of the tomato group.

Multiple comparisons were conducted between the iron concentrations of the treatment groups on day 0 and day 40. The iron concentrations on day 0 were significantly higher (*p*‐value <.05) in the tomato treatment group than that of the SMM and the SMM+Nitrate treatment groups. However, no significant (*p*‐value >.05) differences were found on day 0 between SMM and SMM+Nitrate treatment groups. These results indicate that the iron layer of the metal package was immediately affected by the ingredients in the tomato. After 40 days of storage, significance was found among those three groups (*p*‐value <.05). The samples from the SMM+Nitrate treatment group had the highest average concentration of iron (450.218 ppb), while those of the SMM group had the lowest average concentration (214.015 ppb).

The tin and iron migration from the metal surface of the package to the content was associated with visible signs of corrosion in the sample cans during the storage period. Overall, the results from the ICP‐MS study showed that the effect of the SMM on the corrosion in the BPA‐free coated tin‐plate metal cans was significant. This was concluded because the ICP‐MS results showed that the SMM was associated with an increase in the level of iron migration from the walls of the cans to the packaged tomato products. The level of iron migration was even higher in the presence of the nitrates. At the same time, neither the SMM alone nor the combination of SMM with nitrate had a significant impact on the migration of tin. The corrosion occurring in the canned tomatoes affected both the tin‐plated and the iron layers of the metal package. Thus, both the SMM and nitrate may have synergistically contributed to the corrosion mechanism of iron but not tin. Therefore, other compounds in the tomatoes may have contributed to the migration of tin from the package. Corrosion resulting in the migration of iron and tin into the contents of the cans was observed in other shelf‐life studies with tomato products (Ninčević Grassino et al., 2009; Perring & Basic‐Dvorzak, 2002). Furthermore, the literature supports findings of nitrate at low concentrations in tomatoes and that they resulted in metal migration due to corrosion. In one study, nitrate as low as 50 ppm resulted in the removal of 50% of the tin in canned acidified solutions with nitrate (Olivares et al., 2010). Another study showed that canned tomatoes spiked with nitrate experienced an increase in corrosion and a resultant decrease in the nitrate content in the packaged solution. This study concluded that nitrate in tomatoes works synergistically with oxygen, organic acid, and chloride to cause delamination and detinning in lacquered cans (Albu‐Yaron & Feigin, 1992). While these studies showed that corrosion occurred in cans containing nitrates, acids, and other ingredients, the studies did not report the effect of the individual ingredients on corrosion at concentrations normally found in tomatoes.

## 4 CONCLUSION

This study showed that dimethyl sulfide was one of the highest produced volatile compounds that formed during the retort process of the canned tomatoes. Its formation was due to the thermal degradation of methyl methionine. The SIFT‐MS analysis demonstrated that dimethyl sulfide had significant retention in the lining of the cans after the retorting process. The interaction of the tomato compounds with the polymeric lining resulted in the formation of breaches which created avenues for corrosive compounds to diffuse and react with the base metal, and then initiate corrosion. This corrosion reaction caused tin and iron compounds to migrate from the metal surface of the package into the tomato product. Additionally, this study demonstrated an investigative approach to better understand how compounds within a food product can interact with the package and cause a reduction in the shelf life of the product.

## CONFLICT OF INTEREST

The authors declare that they do not have any conflict of interest.

## AUTHORS CONTRIBUTION

**Elliot Dhuey:** Data curation (equal); Formal analysis (equal); Writing‐original draft (equal). **Hardy Castada:** Formal analysis (supporting); Investigation (supporting); Methodology (supporting); Writing‐review & editing (supporting). **Sheryl Barringer:** Software (supporting); Validation (supporting); Writing‐review & editing (supporting). **Jojo Joseph:** Data curation (equal); Formal analysis (equal); Methodology (supporting). **Christopher Hadad:** Formal analysis (supporting); Funding acquisition (supporting); Investigation (supporting); Methodology (supporting); Project administration (supporting); Resources (supporting); Visualization (supporting); Writing‐review & editing (supporting). **Ken Ruffley:** Conceptualization (supporting); Funding acquisition (supporting); Investigation (supporting); Project administration (supporting); Resources (equal); Validation (supporting); Visualization (equal). **Melvin A. Pascall:** Conceptualization (lead); Data curation (equal); Funding acquisition (lead); Methodology (equal); Project administration (lead); Supervision (lead); Validation (equal); Visualization (lead); Writing‐review & editing (lead).

## STUDIES INVOLVING ANIMAL AND HUMAN SUBJECTS

This study did not involve any human or animal testing.

## INFORMED CONSENT

Written informed consent was obtained from all study participants prior to submitting the manuscript for publication.

## Supporting information

Table S1

Table S2

Table S3

Table S4

Table S5

Table S6

Table S7

## Data Availability

The data that support the findings of this study are available from the corresponding author upon reasonable request.
