# Supplementary material for: Heat‐induced compounds development in processed tomato and their influence on corrosion initiation in metal food cans
Source: Food Sci Nutr. 2021 Jun 27;9(8):4134–45. doi: 10.1002/fsn3.2376 (PMC8358360; doi:10.1002/fsn3.2376)
Supplement: Supplementary file 1 — Table S1 [file FSN3-9-4134-s007.docx]

**Supplemental Table 1. Various treatment groups and the ingredients used in the experiment.**

| **Ingredients** | **Treatment groups** | | | | | | | | |
| --- | --- | --- | --- | --- | --- | --- | --- | --- | --- |
|  | **Tomato**  **(Unprocessed)** | **Tomato (Processed)** | **Sodium Chloride** | **Citric Acid** | **Calcium Chloride** | **Methionine** | **Nitrate** | **SMM** | **Nit+SMM** |
| Diced Tomato | X | X |  |  |  |  |  |  |  |
| Tomato Juice | X | X |  |  |  |  |  |  |  |
| Sodium Chloride | X | X | X |  |  | X | X |  |  |
| Citric Acid, Anhydrous | X | X | X | X | X | X | X | X | X |
| Calcium Chloride | X | X |  |  | X |  |  |  |  |
| Sodium Nitrate |  |  |  |  |  |  | X |  | X |
| Methionine |  |  |  |  |  | X | X |  |  |
| Distilled Water |  |  | X | X | X | X | X | X | X |
| S-Methyl Methionine |  |  |  |  |  |  |  | X | X |
