# Supplementary material for: Heat‐induced compounds development in processed tomato and their influence on corrosion initiation in metal food cans
Source: Food Sci Nutr. 2021 Jun 27;9(8):4134–45. doi: 10.1002/fsn3.2376 (PMC8358360; doi:10.1002/fsn3.2376)
Supplement: Supplementary file 2 — Table S2 [file FSN3-9-4134-s006.docx]

Supplemental Table 2. Selected volatiles, reagent ions and mass-to-charge ratio of products.

| **Volatile Compounds** | **Formula** | **m/z** | **Reagent** | **Reference** |
| --- | --- | --- | --- | --- |
| **Sulfurs** |  |  |  |  |
| dimethyl disulfide | C_2_H_6_S_2_ | 95  94  94 | H_3_O^+^  NO^+^  O_2_^+^ | (Castada et al., 2015) [14] |
| dimethyl sulfide | C_2_H_6_S | 62  62 | NO^+^  O_2_^+^ | (Castada et al., 2015) [14] |
| dimethyl trisulfide | C_2_H_6_S_3_ | 126  126 | NO^+^  O_2_^+^ | (Castada et al., 2015) [14] |
| 2-isobutylthiazole | C_7_H_11_NS | 142  99 | H_3_O^+^  O_2_^+^ | (Xu & Barringer, 2010) [15] |
| methyl mercaptan | CH_4_S | 49  48 | H_3_O^+^  O_2_^+^ | (Španěl & Smith, 1999) [16] |
| 1-propanethiol | C_3_H_8_S | 76+106 | NO^+^ | (Španěl & Smith, 1999) [16] |
| **Acids** |  |  |  |  |
| acetic acid | CH_3_COOH | 90+108 | NO^+^ | (Castada et al., 2015) [14] |
| butanoic acid | C_4_H_8_O_2_ | 89  118 | H_3_O^+^  NO^+^ | (Castada et al., 2015) [14] |
| hexanoic acid | C_6_H_12_O_2_ | 146 | NO^+^ | (Olivares et al., 2010) [17] |
| hexyl acetate | C_8_H_16_O_2_ | 174 | NO^+^ | (Španěl & Smith, 1999) [16] |
| **Others** |  |  |  |  |
| Ammonia | NH_3_ | 18+36 | H_3_O^+^ | (Castada et al., 2015) [14] |
| (E)-2-hexenal | C_6_H_10_O | 97 | NO^+^ | (Xu & Barringer, 2010) [15] |
| (E)-2-octenal | C_8_H_14_O | 125+156 | NO^+^ | (Xu & Barringer, 2010) [15] |
| (E)-2-pentenal | C_5_H_8_O | 83 | NO^+^ | (Xu & Barringer, 2010) [15] |
| furfural | C_5_H_4_O_2_ | 96  96 | NO^+^  O_2_^+^ | (Španěl and Smith 1999) [16] |
| hexanal | C_6_H_12_O | 101+119+137  99 | H_3_O^+^  NO^+^ | (Xu & Barringer, 2010) [15] |
| phenylacetaldehyde | C_8_H_8_O | 121+139+157  120 | H_3_O^+^  NO^+^ | (Xu & Barringer, 2010); [15] (Castada et al., 2015) [14] |
| acetaldehyde | C_2_H_4_O | 45+81  43+61+79 | H_3_O^+^  NO^+^ | (Xu & Barringer, 2010) [15]; (Castada et al., 2015) [14] |
| ethanol | C_2_H_6_O | 45+63+81 | NO^+^ | (Castada et al., 2015) [14] |
| furaneol | C_6_H_8_O_3_ | 129+147  128  128 | H_3_O^+^  NO^+^  O_2_^+^ | (Castada et al., 2015) [14] |
| methanol | CH_3_OH | 33+51+69 | H_3_O^+^ | (Xu & Barringer, 2010) [15] |
| acetone | C_3_H_6_O | 59  88 | H_3_O^+^  NO^+^ | (Xu & Barringer, 2010) [15] |
