# Supplementary material for: Heat‐induced compounds development in processed tomato and their influence on corrosion initiation in metal food cans
Source: Food Sci Nutr. 2021 Jun 27;9(8):4134–45. doi: 10.1002/fsn3.2376 (PMC8358360; doi:10.1002/fsn3.2376)
Supplement: Supplementary file 3 — Table S3 [file FSN3-9-4134-s002.docx]

Supplemental Table 3. Concentrations (ppb_v_) of selected volatiles in the various treatment groups during storage at 49^O^C.

|  |  | Concentration of analytes in 3 grams of sample in various treatment groups | | | | | | | | | | | | | | |
| --- | --- | --- | --- | --- | --- | --- | --- | --- | --- | --- | --- | --- | --- | --- | --- | --- |
|  |  | Citric Acid | | |  | Sodium Chloride | | |  | Calcium Chloride | | |  | Methionine | | |
|  | Volatile Compounds | Day 0 | Day 20 | Day 40 |  | Day 0 | Day 20 | Day 50 |  | Day 0 | Day 20 | Day 40 |  | Day 0 | Day 20 | Day 50 |
| **Sulfurs** | |  |  |  |  |  |  |  |  |  |  |  |  |  |  |  |
|  | dimethyl disulfide | 0 | 0 | 0 |  | 0 | 0 | 0 |  | 0 | 0 | 0 |  | 2 | 47 | 223 |
|  | dimethyl sulfide | 1 | 1 | 1 |  | 1 | 1 | 1 |  | 1 | 1 | 2 |  | 2 | 2 | 2 |
|  | dimethyl trisulfide | -1 | 0 | 0 |  | -1 | 0 | -1 |  | -1 | 0 | -2 |  | -2 | 1 | 5 |
|  | methyl mercaptan | 0 | 0 | 0 |  | 0 | 0 | 0 |  | 0 | 0 | 0 |  | 1 | 7 | 23 |
|  | 1-propanethiol | 0 | 0 | 0 |  | 0 | 0 | 0 |  | 0 | 0 | 3 |  | 7 | 27 | 19 |
|  | 2-isobutylthiazole | 0 | 0 | 0 |  | 0 | 0 | 0 |  | 0 | 0 | 0 |  | 0 | 0 | 0 |
| **Acids** | |  |  |  |  |  |  |  |  |  |  |  |  |  |  |  |
|  | hexanoic acid | -1 | -1 | -1 |  | -1 | -1 | -1 |  | -1 | -1 | -2 |  | -3 | -2 | -2 |
|  | hexyl acetate | -1 | -1 | -1 |  | -1 | -1 | -2 |  | -2 | -1 | 0 |  | -3 | -1 | 0 |
|  | butanoic acid | 0 | -1 | -1 |  | 0 | -1 | 0 |  | 0 | 0 | 0 |  | -1 | 0 | 2 |
|  | acetic acid | -3 | 0 | -1 |  | -4 | 1 | 2 |  | -3 | 1 | 8 |  | -11 | 5 | 18 |
| **Others** | |  |  |  |  |  |  |  |  |  |  |  |  |  |  |  |
|  | Methanol | 66 | 60 | 58 |  | 67 | 50 | 44 |  | 60 | 58 | 86 |  | 96 | 108 | 105 |
|  | Ethanol | 57 | 66 | 64 |  | 29 | 55 | 62 |  | 63 | 76 | 219 |  | 71 | 305 | 321 |
|  | Furaneol | 0 | 0 | 0 |  | 0 | 0 | 0 |  | 0 | 0 | 1 |  | -1 | 0 | 0 |
|  | Furfural | 0 | 0 | 0 |  | 0 | 0 | 0 |  | 0 | 0 | 0 |  | 0 | 5 | 20 |
|  | Hexanal | 1 | 1 | 1 |  | 1 | 1 | 0 |  | 2 | 1 | 0 |  | 1 | 1 | 1 |
|  | Phenylacetaldehyde | -1 | 0 | 0 |  | -1 | 0 | 0 |  | 0 | 0 | -1 |  | -11 | -3 | -3 |
|  | (E)-2-hexenal | 0 | 0 | 0 |  | 0 | 0 | 0 |  | 0 | 0 | 0 |  | 0 | 0 | 0 |
|  | (E)-2-octenal | 0 | 0 | 0 |  | 0 | 0 | 0 |  | 0 | 0 | 0 |  | -6 | 0 | 0 |
|  | (E)-2-pentenal | 0 | 0 | 0 |  | 0 | 0 | 0 |  | 0 | 0 | 0 |  | 0 | 0 | 0 |
|  | Acetaldehyde | 16 | 32 | 36 |  | 17 | 35 | 40 |  | 18 | 51 | 108 |  | 18 | 72 | 141 |
|  | Acetone | 92 | 210 | 253 |  | 86 | 1,000 | 1,196 |  | 72 | 703 | 1887 |  | 83 | 490 | 1220 |
|  | Ammonia | 0 | 3 | 2 |  | 3 | 1 | 3 |  | 2 | 6 | 14 |  | -19 | -9 | -10 |

*Values expressed as the mean of 2 batches by 3 replicates per batch
