# Supplementary material for: Heat‐induced compounds development in processed tomato and their influence on corrosion initiation in metal food cans
Source: Food Sci Nutr. 2021 Jun 27;9(8):4134–45. doi: 10.1002/fsn3.2376 (PMC8358360; doi:10.1002/fsn3.2376)
Supplement: Supplementary file 4 — Table S4 [file FSN3-9-4134-s001.docx]

Supplemental Table 4. Concentrations (ppb_v_) of selected volatiles in the nitrate treatment group during storage at 49^O^C.

|  |  | Concentration of analytes in 3 grams of Nitrate treatment groups (ppb_v_) | | | | | | | | |
| --- | --- | --- | --- | --- | --- | --- | --- | --- | --- | --- |
|  | Volatile Compounds |  | Day 0 | Day 3 | Day 6 | Day 10 | Day 20 | Day 30 | Day 40 | Day 50 |
| **Sulfurs** | |  |  |  |  |  |  |  |  |  |
|  | dimethyl disulfide |  | 4 | 0 | 4 | 4 | 37 | 144 | 189 | 132 |
|  | dimethyl sulfide |  | 2 | 1 | 2 | 1 | 2 | 2 | 2 | 1 |
|  | dimethyl trisulfide |  | -2 | -1 | 0 | 0 | 2 | 7 | 9 | 5 |
|  | methyl mercaptan |  | 2 | 0 | 3 | 3 | 8 | 19 | 28 | 28 |
|  | 1-propanethiol |  | 8 | 4 | 13 | 14 | 24 | 12 | 11 | 13 |
|  | 2-isobutylthiazole |  | 0 | 0 | 0 | 0 | 0 | 0 | 0 | 0 |
| **Acids** | |  |  |  |  |  |  |  |  |  |
|  | hexanoic acid |  | -3 | 0 | -1 | -1 | -1 | -2 | -1 | 0 |
|  | hexyl acetate |  | -4 | -2 | -1 | -1 | -1 | -2 | -2 | 0 |
|  | butanoic acid |  | -1 | 0 | -1 | -1 | -1 | -1 | -1 | -1 |
|  | acetic acid |  | -11 | -4 | -2 | -3 | 3 | 9 | 14 | 11 |
| **Others** | |  |  |  |  |  |  |  |  |  |
|  | Methanol |  | 102 | 67 | 75 | 70 | 81 | 81 | 80 | 72 |
|  | Ethanol |  | 79 | 27 | 72 | 77 | 9 | 41 | 30 | 95 |
|  | Furaneol |  | -1 | 0 | 0 | 0 | 0 | 0 | 0 | 0 |
|  | Furfural |  | 0 | 0 | 0 | 0 | 4 | 14 | 17 | 12 |
|  | Hexanal |  | 1 | 1 | 1 | 1 | 2 | 1 | 1 | 1 |
|  | phenylacetaldehyde |  | -14 | -1 | 0 | 0 | 0 | 1 | 1 | 0 |
|  | (E)-2-hexenal |  | 0 | 0 | 0 | 0 | 0 | 0 | 1 | 1 |
|  | (E)-2-octenal |  | -6 | 0 | -1 | -1 | 0 | 0 | 0 | 0 |
|  | (E)-2-pentenal |  | 0 | 0 | 0 | 1 | 0 | 0 | 0 | 0 |
|  | Acetaldehyde |  | 14 | 18 | 25 | 25 | 54 | 108 | 117 | 85 |
|  | Acetone |  | 84 | 48 | 148 | 132 | 348 | 817 | 1,099 | 726 |
|  | Ammonia |  | -15 | 3 | 1 | 0 | -4 | -5 | -5 | -8 |

*Values expressed as the mean of 2 batches by 3 replicates per batch.
