# Supplementary material for: Heat‐induced compounds development in processed tomato and their influence on corrosion initiation in metal food cans
Source: Food Sci Nutr. 2021 Jun 27;9(8):4134–45. doi: 10.1002/fsn3.2376 (PMC8358360; doi:10.1002/fsn3.2376)
Supplement: Supplementary file 5 — Table S5 [file FSN3-9-4134-s003.docx]

Supplemental Table 5. Concentrations (ppb_v_) of selected volatile compounds in the SMM treatment group during storage at 49^O^C.

|  |  | Concentration of analytes in 3 grams of SMM sample (ppb_v_) | | | | | | | |
| --- | --- | --- | --- | --- | --- | --- | --- | --- | --- |
|  | Volatile Compounds | Day 0 | Day 3 | Day 6 | Day 10 | Day 20 | Day 30 | Day 40 | Day 50 |
| **Sulfurs** | |  |  |  |  |  |  |  |  |
|  | dimethyl disulfide | 8 | 8 | 21 | 9 | 49 | 91 | 65 | 46 |
|  | dimethyl sulfide | 3390 | 3129 | 2261 | 2495 | 1028 | 470 | 14 | 19 |
|  | dimethyl trisulfide | 1 | 1 | 4 | 1 | 2 | 5 | 2 | 1 |
|  | methyl mercaptan | 1 | 1 | 3 | 1 | 4 | 5 | 2 | 2 |
|  | 1-propanethiol | 1 | 2 | 5 | 2 | 5 | 4 | 0 | 0 |
|  | 2-isobutylthiazole | 0 | 0 | 0 | 0 | 0 | 0 | 0 | 0 |
| **Acids** | |  |  |  |  |  |  |  |  |
|  | hexanoic acid | 0 | 0 | 0 | 0 | 0 | 0 | 0 | 0 |
|  | hexyl acetate | 0 | 0 | 0 | -1 | -1 | -1 | -1 | -1 |
|  | butanoic acid | 0 | 0 | 0 | 1 | 1 | 1 | 0 | 1 |
|  | acetic acid | -1 | -2 | 0 | -3 | 0 | 5 | 5 | 4 |
| **Others** | |  |  |  |  |  |  |  |  |
|  | methanol | 79 | 77 | 80 | 65 | 81 | 79 | 86 | 88 |
|  | ethanol | 294 | 283 | 222 | 230 | 146 | 83 | 54 | 67 |
|  | furaneol | 0 | 0 | 0 | 0 | 0 | 0 | 0 | 0 |
|  | furfural | 0 | 0 | 2 | 0 | 4 | 6 | 5 | 3 |
|  | hexanal | 1 | 1 | 0 | 0 | 0 | 1 | 1 | 1 |
|  | phenylacetaldehyde | 0 | 0 | 0 | 0 | 0 | 0 | 0 | 0 |
|  | (E)-2-hexenal | 0 | 0 | 0 | 0 | 0 | 0 | 0 | 0 |
|  | (E)-2-octenal | 0 | 0 | 0 | 0 | 0 | 0 | 0 | 0 |
|  | (E)-2-pentenal | 0 | 1 | 0 | 12 | 0 | 41 | 0 | 0 |
|  | acetaldehyde | 25 | 28 | 34 | 26 | 42 | 52 | 74 | 71 |
|  | acetone | 161 | 228 | 408 | 222 | 583 | 914 | 1105 | 1052 |
|  | ammonia | -3 | -6 | -5 | -8 | -4 | -3 | -4 | -6 |

*Values expressed as the mean of 2 batches by 3 replicates per batch.
