# Supplementary material for: Heat‐induced compounds development in processed tomato and their influence on corrosion initiation in metal food cans
Source: Food Sci Nutr. 2021 Jun 27;9(8):4134–45. doi: 10.1002/fsn3.2376 (PMC8358360; doi:10.1002/fsn3.2376)
Supplement: Supplementary file 6 — Table S6 [file FSN3-9-4134-s004.docx]

Supplemental Table 6. Changes in the concentrations (ppb_v_) of selected volatile compounds of the can lining processed with tomatoes during storage at 49^O^C.

|  |  | Concentration of analytes in 6 mg of can lining (ppb_v_) processed in tomato | | | | | | | |
| --- | --- | --- | --- | --- | --- | --- | --- | --- | --- |
| Volatile Compounds | | Day 0 | Day 3 | Day 6 | Day 10 | Day 20 | Day 30 | Day 40 | Day 50 |
| **Sulfurs** | |  |  |  |  |  |  |  |  |
|  | dimethyl disulfide | 0 | 1 | 1 | 0 | 0 | 0 | 0 | 0 |
|  | dimethyl sulfide | 62 | 165 | 157 | 166 | 193 | 145 | 164 | 103 |
|  | dimethyl trisulfide | 3 | 5 | 5 | 4 | 5 | 4 | 5 | 3 |
|  | methyl mercaptan | 0 | 1 | 1 | 1 | 1 | 1 | 1 | 0 |
|  | 1-propanethiol | 1 | 2 | 1 | 1 | 1 | 1 | 1 | 0 |
|  | 2-isobutylthiazole | 0 | 1 | 1 | 1 | 1 | 0 | 1 | 0 |
| **Acids** | |  |  |  |  |  |  |  |  |
|  | hexanoic acid | 0 | 0 | 0 | -1 | 1 | -1 | -1 | -1 |
|  | hexyl acetate | 0 | 1 | 0 | 0 | 0 | -3 | -3 | -3 |
|  | butanoic acid | -1 | 1 | 1 | 1 | 1 | 1 | 1 | 0 |
|  | acetic acid | -3 | 0 | -3 | -3 | -3 | -1 | 0 | -3 |
| **Others** | |  |  |  |  |  |  |  |  |
|  | methanol | 44 | 171 | 370 | 337 | 377 | 115 | 146 | 83 |
|  | ethanol | 78 | 159 | 251 | 260 | 279 | 82 | 94 | 61 |
|  | furaneol | 0 | 0 | 1 | 0 | 1 | 0 | 0 | 0 |
|  | furfural | 1 | 2 | 2 | 3 | 4 | 5 | 6 | 6 |
|  | hexanal | 2 | 4 | 4 | 4 | 4 | 5 | 4 | 3 |
|  | phenylacetaldehyde | 1 | 1 | 0 | 0 | 1 | 1 | 1 | 0 |
|  | (E)-2-hexenal | 1 | 2 | 1 | 1 | 1 | 1 | 1 | 0 |
|  | (E)-2-octenal | 0 | 0 | 0 | 0 | 0 | 0 | 0 | 0 |
|  | (E)-2-pentenal | 1 | 1 | 1 | 1 | 2 | 1 | 1 | 1 |
|  | acetaldehyde | 11 | 27 | 20 | 16 | 22 | 13 | 17 | 18 |
|  | acetone | 6 | 22 | 23 | 13 | 20 | 7 | 12 | 7 |
|  | ammonia | 16 | 42 | 27 | 18 | 31 | -2 | -2 | -18 |

*Values expressed as the mean of 2 batches by 2 replicates per batch.
