# Supplementary material for: Heat‐induced compounds development in processed tomato and their influence on corrosion initiation in metal food cans
Source: Food Sci Nutr. 2021 Jun 27;9(8):4134–45. doi: 10.1002/fsn3.2376 (PMC8358360; doi:10.1002/fsn3.2376)
Supplement: Supplementary file 7 — Table S7 [file FSN3-9-4134-s005.docx]

Supplemental Table 7. Concentrations (ppb_v_) of selected volatile compounds in the can lining processed with SMM treatment group during storage at 49^O^C.

|  |  | Concentration of analytes in 6 mg of can lining processed with SMM (ppb_v_) | | | | | | | |
| --- | --- | --- | --- | --- | --- | --- | --- | --- | --- |
|  | Volatile Compounds | Day 0 | Day 3 | Day 6 | Day 10 | Day 20 | Day 30 | Day 40 | Day 50 |
| **Sulfurs** | |  |  |  |  |  |  |  |  |
|  | dimethyl disulfide | 1 | 0 | 4 | 1 | 14 | 19 | 22 | 12 |
|  | dimethyl sulfide | 156 | 95 | 92 | 89 | 47 | 16 | 1 | 1 |
|  | dimethyl trisulfide | 0 | -2 | -1 | -2 | -1 | -1 | -1 | -1 |
|  | methyl mercaptan | 1 | 0 | 2 | 1 | 2 | 2 | 2 | 2 |
|  | 1-propanethiol | -4 | -7 | -7 | -9 | -9 | -9 | -9 | -9 |
|  | 2-isobutylthiazole | 0 | -1 | 0 | 0 | 0 | -1 | -1 | 0 |
| **Acids** | |  |  |  |  |  |  |  |  |
|  | hexanoic acid | 3 | -2 | -2 | -3 | -2 | -2 | -2 | -2 |
|  | hexyl acetate | -2 | -3 | -2 | -4 | -3 | -3 | -3 | -4 |
|  | butanoic acid | -3 | -3 | -3 | -4 | -3 | -3 | -3 | -3 |
|  | acetic acid | -7 | -10 | -9 | -12 | -10 | -10 | -9 | -10 |
| **Others** | |  |  |  |  |  |  |  |  |
|  | methanol | 10 | 0 | 34 | 27 | 37 | 43 | 34 | 43 |
|  | ethanol | 302 | 300 | 462 | 365 | 613 | 672 | 749 | 829 |
|  | furaneol | -1 | -2 | -1 | -1 | -1 | -1 | -1 | -1 |
|  | furfural | 0 | 0 | 0 | 0 | 2 | 2 | 3 | 1 |
|  | hexanal | -1 | -2 | -1 | -2 | -2 | -2 | -2 | -2 |
|  | phenylacetaldehyde | -2 | -4 | -4 | -5 | -5 | -4 | -4 | -4 |
|  | (E)-2-hexenal | 0 | -1 | 0 | -1 | -1 | -1 | -1 | -1 |
|  | (E)-2-octenal | 0 | 0 | 0 | -1 | -1 | 0 | 0 | -1 |
|  | (E)-2-pentenal | 0 | 69 | 0 | -1 | 0 | 0 | 0 | 0 |
|  | acetaldehyde | 17 | 6 | 12 | 3 | 10 | 8 | 14 | 13 |
|  | acetone | -4 | -3 | -4 | -9 | -3 | 0 | 5 | 1 |
|  | ammonia | -44 | -51 | -47 | -59 | -51 | -52 | -47 | -48 |

*Values expressed as the mean of 2 batches by 2 replicates per batch.
